# Extended-spectrum β-lactamases and virulence factors in uropathogenic *Escherichia coli* in nursing homes in Lima, Peru

**DOI:** 10.17843/rpmesp.2022.391.8580

**Published:** 2022-03-24

**Authors:** Arturo Octavio Gonzales-Rodriguez, Stefany Fiorella Infante Varillas, Carlos Ignacio Reyes-Farias, Cesar Enrique Ladines Fajardo, Edgar Gonzales Escalante

**Affiliations:** 1 Facultad de Medicina Humana, Universidad de Piura, Lima, Peru. Universidad Nacional de Piura Facultad de Medicina Humana Universidad de Piura Lima Peru; 2 Instituto de Investigaciones en Bacteriología y Virología Molecular (IBaViM), Facultad de Farmacia y Bioquímica, Universidad de Buenos Aires, Buenos Aires, Argentina. Universidad de Buenos Aires Instituto de Investigaciones en Bacteriología y Virología Molecular (IBaViM) Facultad de Farmacia y Bioquímica Universidad de Buenos Aires Buenos Aires Argentina

**Keywords:** Urinary infections, Antibacterials, Beta-Lactam Resistance, Enterobacteriaceae Infections, Nursing Homes, Uropathogenic, *Escherichia coli*, Virulence Factors, Urinary Tract Infections, ß-lactamases

## Abstract

Nursing homes are institutions with high prevalence of urinary tract infections caused by ESBL-producing *E. coli* with several virulence factors. The aim of this study was to determine the frequency of the *bla*_CTX-M _gene and eight virulence genes in 35 ESBL-producing uropathogenic *E. coli* from six nursing homes in Peru during 2018. Of the *E. coli* samples, 57.1% (20/35) were carriers of the *bla*_CTX-M _gene. Furthermore, we obtained frequencies of 46% (15/35) and 37% (13/35) for *hly-alpha* and *cnf-1*, respectively; we also found high presence of the *iuc*C (63%, 22/35), *aer* (94%, 33/35) and *chu*A genes (94%, 33/34) as well as a frequency of 46% (16/35) and 91% (32/34) for the *pap GII* and *nan*A genes, respectively. The *bla*_CTX-M_ gene is predominant and a high frequency of exotoxins gives it a competitive advantage for spreading into the bloodstream.

## INTRODUCTION

Nursing homes are long-term care institutions with a high prevalence of infectious diseases ^1^; urinary tract infection (UTI), whose main etiological agent is *Escherichia coli*, is the most frequent infection in this type of institution ^1^. Likewise, between 20 to 40% of bacteremias in the elderly population are caused by bacteria that ascend through the urinary tract; *E. coli* and *Proteus mirabilis* are the most common microbial agents ^2^.

Nursing home residents have a high probability of colonization and infections by multidrug-resistant *E. coli* ^3^. In addition, the high recurrence of UTI and inappropriate treatment of asymptomatic bacteriuria increases the use of antibiotics, which leads to the selection of resistant bacteria ^1^, with an increase in the mortality rate of residents ^2^.

ß-lactam antibiotics represent approximately 50% of the antibiotics prescribed worldwide ^4^; however, their therapeutic efficiency has decreased due to the rapid selection of resistance mechanisms. Extended-spectrum ß-lactamases (ESBLs) represent the most important mechanism for resistance to third-generation cephalosporins in *E. coli*. CTX-M enzymes, due to their high propagation efficiency, have displaced SHV and TEM type ß-lactamases as the most prevalent worldwide ^4^. CTX-M has a heterogeneous lineage that includes six groups (CTX-M-1, CTX-M-2, CTX-M-8, CTX-M-9, CTX-M-25 and KLUC) that are different from each other by 10% or more amino acid residues ^4^. ESBL-producing *E. coli. *are highly frequent in Peru; in 2011, a multinational surveillance program that evaluated eleven Latin American countries found that 54.0% of *E. coli.* were ESBL-producing ^5^.

On the other hand, although the dissemination of CTX-M type ESBL in uropathogenic *E. coli* (UPEC) is a global problem that decreases therapeutic options in patients, the severity of infection depends on the pathogenic capacity of UPEC ^6^. UPEC have adherence factors (e.g. *Pap G II*) that allow them to successfully initiate infection and migrate to the upper regions of the urinary tract, aggravating the patient’s condition ^6^. Likewise, UPEC produce siderophore systems (e.g. *ChuA, Aer*) and exotoxins (e.g. α*-Hly, TcpC, Cnf-1*) that together constitute a protein system that allow them to evade and/or affect the immune system to the detriment of the patient’s health ^6^. In bacteremias caused by an UTI, UPEC require the expression of proteins that allow them to adapt to the environmental conditions of the blood tissue (e.g. *nanA*) ^7^.

To our knowledge, no studies that describe the frequency of CTX-M type ESBL-producing UPEC in nursing homes have been published in Peru. The population of these nursing homes is susceptible to UTI and systemic complications; therefore, the aim of this study was to determine the frequency of virulence genes and the presence of the *bla*_CTX-M _gene in UPEC isolated from residents of nursing homes located in Metropolitan Lima.

**KEY MESSAGES**

Motivation for the study: Nursing homes deserve special epidemiological attention because of the high prevalence of urinary tract infections due to multidrug-resistant *Escherichia coli* and the high risk of urosepsis.

Main findings: The *bla*_CTX-M_ gene was carried by 57.1% of the ESBL-producing *E. coli *and 70% belonged to the *bla*_CTX-M-group 2_ gene. Additionally, we obtained a frequency of 46% and 37% for *hly-alpha* and *cnf-1*, respectively; as well as 46% and 91% for the *pap GII* and *nanA* gene, respectively.

Implications: This is the first report of resistant genes associated with ESBL in uropathogenic *E. coli* identified in nursing homes in Peru, which is of great relevance because it represents a threat to the health of the elderly population.

## THE STUDY

### Study design and population

Observational descriptive study conducted in 2018, of 35 random non-duplicate isolates of ESBL-producing *E. coli* obtained from urine samples of older adult residents of six private nursing homes in Metropolitan Lima in Peru.

### Microbiological study

Bacteria were identified by using conventional biochemical tests (triple sugar iron agar, lysine iron agar, citrate agar, mobility-indol-orinithine medium and methyl red/Voges-Proskauer) and confirmed molecularly, by polymerase chain reaction (PCR), through the amplification of the *uspA* gene.

### Susceptibility testing

Presence of ESBL-producing *E. coli *was confirmed by the diffusion disc method specified in Clinical & Laboratory Standards Institute (CLSI) document M02-13 ^8^; in addition, antimicrobial susceptibility was determined by the diffusion disc method according to CLSI guidelines ^8^, using *E. coli* ATCC 25922 as a quality control. Antibiotic discs with the following antibiotics were included: piperacillin/tazobactam 100/10 µg (PTZ); amoxicillin/clavulanic acid 20/10 µg (AMC); cefotaxime 30 µg (CTX); ceftazidime 30 µg (CAZ); cefepime 30 µg (FEP); cefoxitin 30 µg (FOX); aztreonam 30 µg (AZM); meropenem 10 µg (MEM); imipenem 10 µg (IMP); amikacin 30 µg (AK); gentamicin 10 µg (GM); ciprofloxacin 5 µg (CIP); nitrofurantoin 300 µg (NIT) and trimethoprim/sulfamethoxazole 1.25/23.75 µg (SXT).

### Detection of virulence and resistance genes

Bacterial DNA was extracted using the DNA Purification kit Gene-JetGenomic (ThermoScientific), following the manufacturer’s recommendations. Eight virulence genes were identified: *aer, hly, cnf-1, chuA, TcpC, nanA, pap GII, iucC* ^9^ ^,^ ^10^, as well as three ESBL-associated resistance genes: *bla*_CTX-M_, *bla*_SHV_ and *bla*_TEM_ ^11^. In addition, different groups of isolates carrying *bla*_CTX-M_ (1, 2, 8 and 9) were detected ^12^. PCR conditions were carried out in a final volume of 30 μl containing 1X of MaximoTaq DNA (GeneON) as well as 0.5 uM of each of the primers and 1.5 mM MgCl_2_. The volume of incorporated DNA was 2.0 μL. The Labnet, Multigene optimax thermal cycler was programmed with the following parameters: initial denaturation at 94 °C for three minutes; denaturation at 94 °C for 30 seconds; extension at 72 °C for 30 seconds; in 30 reaction cycles and final extension at 72 °C for three minutes. Table 1 shows the sequence of the primers used, the size of the amplification products, the hybridization temperature and the reference of the study. The amplified DNA fragments were separated by 1% agarose gel electrophoresis for 50 min at 110 volts. Finally, Runsafe loading buffer (GeneON) was used for UV development.

**Table 1:** Virulence and resistance genes, amplification characteristics.

Gene	Primer sequence (5’ - 3’)	Product (bp)	Hybridization temperature (°C)
Virulence genes			
α-hemolysin (α-*hly*)	AACAAGGATAAGCACTGTTCTGGCT	1177	63
	ACCATATAAGCGGTCATTCCCGTCA		
*chuA*	GACGAACCAACGGTCAGGAT	279	55
	TGCCGCCAGTACCAAAGACA		
Aerobactin synthesis (*aer*)	TACCGGATTGTCATATGCAGACCGT	602	63
	AATATCTTCCTCCAGTCCGGAGA AG		
Aerobactin synthesis (*iucC*)	CTCGAATTCACTGGGATTTGGTCAACC	1701	62
	CTCTCTAGAATTCCTGAGTTACCAGCC		
Cytotoxic necrotizing factor (*cnf1*)	AAGATGGAGTTTCCTATGCAGGAG	498	61
	CATTCAGAGTCCTGCC CTCATTATT		
P - fimbriae (*pap*)-* alelo II*	GGGATGAGCGGGCCTTTGAT	190	65
	CGGGCCCCCAAGTAACTCG		
*nanA*	ACCGGTGAGGGGAAATAAAC	216	59
	GGTGAGTACCAGGGCGATTA		
*tcpC*	GGCAACAATATGTATAATATCCT	386	51
	GCCCAGTCTATTTCTGCTAAAGA		
Resistance genes			
*bla*SHV	ATGCGTTATATTCGCCTGTG	544	58
	GTTAGCGTTGCCAGTGCTCG		
*bla*TEM	ATAAAATTCTTGAAGACGAAA	1080	54
	GACAGTTACCAATGCTTAATC		
*bla*CTX-M	TTTGCGATGTGCAGTACCACTAA	865	60
	CGATATCGTTGGTGGTGCCAT		
*bla*CTX-M-group 1	ATGGTTAAAAAATCACTG C	900	55
	GGTGACGATTTTAGCCGC		
*bla*CTX-M-group 2	CGTTAACGGCACGATGAC	404	59
	CGATATCGTTGGTGGTGCCAT		
*bla*CTX-M-group 8	ACGCTCAACACCGCGATC	490	63,3
	CGTGGGTTCTCGGGGATAA		
*bla*CTX-M-group 9	GATTGACCGTATTGGGAGTTT	831	58
	CGGCTGGGTAAAATAGGTCA		

### Statistical analysis

Statistical analysis was carried out with SPSS Statistics for Windows, version 25.0. Armonk, NY: IBM Corp. Qualitative variables were described by using frequency graphs.

### Ethical considerations

This study was approved by the Ethics Committee of the Faculty of Medicine of the Universidad Nacional Mayor de San Marcos. Act 1812 with project code 0013.

## FINDINGS

### Antimicrobial Susceptibility

**Figure 1:**
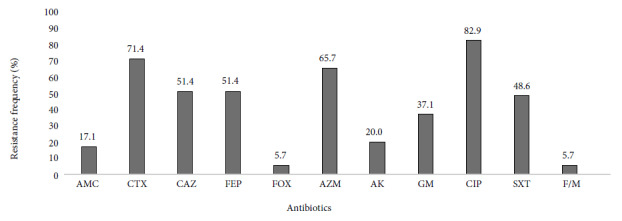
Antimicrobial resistance profile of extended-spectrum ß-lactamase-producing *Escherichia coli* isolates (n=35).

Thirty-five ESBL-producing UPEC from nursing homes were analyzed. Most isolates were resistant to cefotaxime (25/35), there were no carbapenem-resistant isolates, and only two (2/35) isolates were resistant to cefoxitin. In addition to resistance to ß-lactams, we observed that UPEC strains were highly resistant to ciprofloxacin, 82.9% (29/35) of the cases. UPEC resistant to PTZ, MEM and IMP were not found. Figure 1 shows the frequencies of resistance by antibiotic.

### Genotyping of UPEC carrying *bla*_CTX-M_

Of the 35 isolates, 57.1% (20/35) carried the *bla*_CTX-M_ gene, of which 70% (14/20) corresponded to *bla*_CTX-M-group 2_, 40% (8/20) to *bla*_CTX-M-group 1_, 30% (6/20) to *bla*_CTX-M-group 9_ and 20% (4/20) to *bla*_CTX-M-group 8_. On the other hand, 60% (12/20) of the UPEC carrying the *bla*_CTX-M_ gene had two or more allelic variants of the *bla*_CTX-M_ gene in the same bacterium. In addition, there was a higher level of confluence between the genes from *bla*_CTX-M-group 1_ and *bla*_CTX-M-group 2_ (Table 2). The presence of other ß-lactamase-producing genes (*bla*_SHV_ and *bla*_TEM_) was also evaluated in *bla*_CTX-M_ producers and non-producers. Of a substratum of the analyzed UPEC, 48.1% (13/27) did not carry the *bla*_CTX-M_ gene, but did carry the *bla*_SHV_ and/or *bla*_TEM_ genes. Likewise, 51.9% (14/27) of the UPEC presented the *bla*_CTX-M_ gene, in addition to the *bla*_SHV_ and/or *bla*_TEM_ genes. The nucleotide sequences of the allelic variants that would confirm belonging to the ESBL group were not analyzed.

**Table 2:**
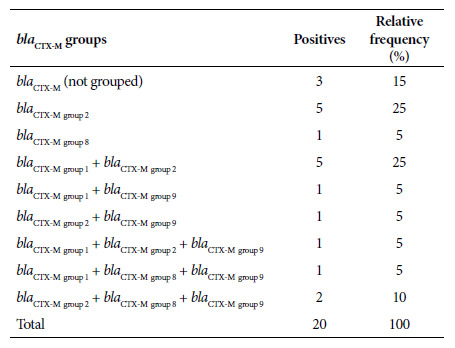
Distribution of blaCTX-M gene clusters in uropathogenic Escherichia coli producing extended-spectrum ß-lactamases

### Genotyping of virulence factors in ESBL-producing UPEC

**Figure 2:**
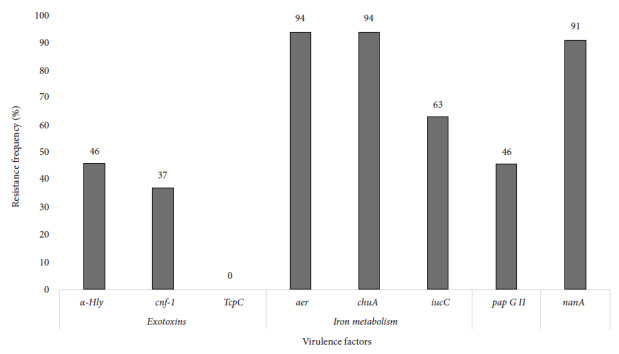
Frequency of virulence genes in uropathogenic *Escherichia coli*.

After analyzing genes related to exotoxins in the 35 ESBL-producing UPEC, we obtained a frequency of 46% (15/35) and 37% (13/35) of *hly-alpha* and *cnf-1*, respectively. *aer* (94%, 33/35) and *chuA* (94%, 33/35) were the most frequent genes associated with iron metabolism. Furthermore, adhesin *pap GII *was found in 46% (16/34) of the isolates and the *nanA* gene in 91% (32/35) (Figure 2).

## DISCUSSION

The results of this study show that 57.1% of the ESBL-producing *Escherichia coli* carried the *bla*_CTX-M _gene, 70% of which belonged to *bla*_CTX-M-group 2_. On the other hand, the genes: *hly-alpha* and *cnf-1*, that code for exotoxins, were detected with a frequency of 46 and 37%, respectively.

Nursing homes are long-stay institutions where the prevalence of UTI caused by multidrug-resistant bacteria is high ^3^. In this study, 57.1% of the UPEC carried the *bla*_CTX-M_ gene. This result is similar to those described by Galván *et al*.^13^ and Arce-Gil *et al*.^11^, who reported a frequency of 54.7% and 51.4%, respectively. However, they differ from those described by Ramirez* et al*. ^14^ and Chavez ^15^ in various hospitals in Lima (70% and 91.7%). The latter demonstrates the great variability in the presence of this gene in health institutions.

Results regarding the *bla*_CTX-M_ subgroups showed that* bla*_CTX-M-group 2_ (14/20) followed by *bla*_CTX-M-group 1_ (8/20) were the most frequent. This differs from what was reported by Chavez ^15^, where *bla*_CTX-M-group 2_ was not detected and *bla*_CTX-M-group 1_ (26/33) had the highest frequency; and by Palma *et al*. ^16^, where *bla*_CTX-M-group 1_ predominated (13/27). However, recent studies indicate that *bla*_CTX-M-group 2_ is still significant in South America ^17^ and a recent review in Brazil identifies *bla*_CTX-M-group 2_ and *bla*_CTX-M-group 1_ as the most prevalent variants in the region ^18^. 

We also detected* bla*_TEM_ (54.3%) and *bla*_SHV_ (51.4%) genes, which were found among UPEC carrying the *bla*_CTX-M_ gene. It is important to note that in 37% of the isolates, only the genes *bla*_TEM_ and/or *bla*_SHV_ were identified, despite the fact that its allelic variant and its belonging to the ESBL group was not determined, this is a strong indicator of its membership. None of the evaluated resistance genes were detected in 5.7% of the isolates, which indicates the presence of other ESBL-type resistance genes that were not analyzed in this study.

Regarding virulence factors, the *pap GII* gene was present in 47.1% of UPEC. These findings differ from the results obtained by Paniagua-Contreras *et al*. ^19^, who reported a 21.1% frequency of the *pap GII* gene in community UPEC in Mexico. We also found a high frequency of genes associated with iron transport, similar to what was reported by Dadi *et al*. ^20^, with 54.5% for the *iucC* gene.

Regarding exotoxins, we obtained a frequency of 44.2% and 38.2% of *hly-alpha* and *cnf-1*, respectively, these results are comparable to those reported by Dadi *et al*. ^20^ who found 50.4% and 29% of *hly-alpha* and *cnf-1*, respectively. On the other hand, in this study we did not find UPEC carrying the *tcpC* gene, in contrast to other studies that have reported prevalence of up to 25% ^10^.

We also found a frequency of 88.2% for the *nanA* gene, which is important for energy production. It has been proposed that the presence of the *nanA* gene creates high competitiveness in UPEC to cause bacteremias, although its role in the pathogenesis of UTI is less important ^6^, the results point to the existence of high risk in residents with bacteremia that develops into sepsis.

This study has some limitations. Although it was carried out in several nursing homes, no clinical information was obtained from the patients, in addition to having a limited number of samples. Moreover, we did not analyze further virulence and resistance genes (other types of ESBL) relevant to the epidemiology of UPEC.

In conclusion, this is the first report of ESBL-producing UPEC in nursing homes in Peru, which shows predominance of the *bla*_CTX-M _gene, 70% of which belong to *bla*_CTX-M-group 2_. Also, we can point out that ESBL-producing UPEC in nursing homes present a high frequency of exotoxins and the *nanA* gene, which gives them an advantage to disseminate into the bloodstream. We hope that these findings will allow strengthening the epidemiological surveillance of multidrug-resistant bacteria and prevent their dissemination.
