# Supplementary material for: Extended-spectrum β-lactamases and virulence factors in uropathogenic Escherichia coli in nursing homes in Lima, Peru
Source: Rev Peru Med Exp Salud Publica. 2022 Mar 24;39(1):98–103. doi: 10.17843/rpmesp.2022.391.8580 (PMC11397600; doi:10.17843/rpmesp.2022.391.8580)
Supplement: Translation

# Translation:  β-lactamasas de espectro extendido y factores de virulencia en *Escherichia coli* uropatógenas en asilos de ancianos en Lima, Perú

**Authors:** Arturo Octavio Gonzales-Rodriguez, Stefany Fiorella Infante Varillas, Carlos Ignacio Reyes-Farias, Cesar Enrique Ladines Fajardo, Edgar Gonzales Escalante

**Affiliations:** 1 Facultad de Medicina Humana, Universidad de Piura, Lima, Perú.; 2 Instituto de Investigaciones en Bacteriología y Virología Molecular (IBaViM), Facultad de Farmacia y Bioquímica, Universidad de Buenos Aires, Buenos Aires, Argentina.

**Keywords:** Infecciones urinarias, Antibacterianos, Resistencia Betalactámica, Infecciones por Enterobacteriaceae, Asilos de Ancianos, *Escherichia coli *, Uropatogénica, Factores de Virulencia, Infecciones Urinarias, ß-lactamasas

## Abstract

Los asilos de ancianos son instituciones con una alta prevalencia de infecciones del tracto urinario ocasionado por *Escherichia coli *productoras de ß-lactamasas de espectro extendido (BLEE), con diversos factores de virulencia. El objetivo del estudio fue determinar la frecuencia del gen *bla*_*CTX-M *_y de ocho genes de virulencia en 35 *E. coli *uropatógenas productoras de BLEE provenientes de seis asilos en Perú, durante el 2018. El 57,1% (20/35) de las *E. coli* fueron portadores del gen *bla*_CTX-M_. Además, se obtuvo una frecuencia del 46% (15/35) y 37% (13/35) de *hly-alfa y cnf-1, *respectivamente; elevada presencia de los genes *iuc*C (63%, 22/35),* aer *(94%, 33/35) *y chu*A (94%, 33/34) y una frecuencia del 46% (16/35) y del 91% (32/34) de los genes *pap GII *y *nanA*, respectivamente. Existe predominancia en la distribución del gen *bla*_CTX-M_, además de una alta frecuencia de exotoxinas que le confieren una ventaja competitiva para diseminarse hacia el torrente sanguíneo.

## INTRODUCCIÓN

Los asilos de ancianos son instituciones de cuidado de largo plazo con una alta prevalencia de enfermedades infecciosas ^1^; la infección del tracto urinario (ITU), cuyo principal agente etiológico es *Escherichia coli*, es la infección más frecuente en este tipo de instituciones ^1^. Asimismo, entre el 20 a 40% de las bacteriemias en ancianos es ocasionada por bacterias que ascienden por el tracto urinario; *E. coli *y *Proteus mirabilis* son los agentes microbianos más comunes ^2^. 

Los residentes de asilos de ancianos presentan una alta probabilidad de colonización y de infecciones por *E. coli *multidrogorresistentes^ (^ ^3^. Además, la alta recurrencia de ITU y el tratamiento inapropiado de bacteriurias asintomáticas incrementa el uso de antibióticos, lo que conlleva a la selección de este tipo de bacterias ^1^, con un aumento de la tasa de mortalidad de los residentes ^2^.

Los antibióticos del grupo ß-lactámico representan aproximadamente el 50% de los antibióticos prescritos en el mundo ^4^; sin embargo, su eficiencia terapéutica ha disminuido debido a la rápida selección de mecanismos de resistencia. Las ß-lactamasas de espectro extendido (BLEE) representan el mecanismo más importante para la resistencia a cefalosporinas de tercera generación en *E. coli*. La enzima denominada CTX-M es la BLEE de mayor predominancia a nivel global debido a su alta eficiencia de propagación desplazando a ß-lactamasas del tipo SHV y TEM en la epidemiología global^ (^ ^4^. La CTX-M tiene un linaje heterogéneo que incluye seis grupos (CTX-M-1, CTX-M-2, CTX-M-8, CTX-M-9, CTX-M-25 y KLUC) que difieren entre sí por una cantidad mayor o igual al 10% de residuos aminoacídicos^ (^ ^4^. En el Perú existe una alta frecuencia de *E. coli *productoras de BLEE; en el 2011, en un programa de vigilancia multinacional en el que se evaluaron once países de América Latina se reportó que el 54,0% de las *E. coli* eran productoras de BLEE^ (^ ^5^. 

Por otro lado, aunque la diseminación de BLEE de tipo CTX-M en *E. coli *uropatógenas (UPEC, por sus siglas en inglés) es un problema global que disminuye las opciones terapéuticas en los pacientes, la severidad de la infección depende de la capacidad patogénica en las UPEC^ (^ ^6^. Las UPEC poseen factores de adherencia (p.ej. *Pap G II*) que le permiten iniciar la infección exitosamente y migrar hacia regiones ascendentes del conducto urinario agravando la condición del paciente ^6^. Asimismo, las UPEC producen sistemas sideróforos (p.ej. *ChuA, Aer*) y exotoxinas (p.ej. *α-Hly, TcpC*, *Cnf-1*) que, en conjunto, constituyen un sistema proteico que les permiten eludir y/o afectar al sistema inmune en detrimento de la salud del paciente^ (^ ^6^. En bacteriemias cuyo punto de origen es una ITU las UPEC requieren de la expresión de proteínas que le permiten adaptarse a las condiciones ambientales del tejido sanguíneo (p.ej.* nanA*)^ (^ ^7^. 

A nuestro conocimiento, no se han publicado en Perú estudios relacionados que hayan descrito la frecuencia de las UPEC productoras de BLEE de tipo CTX-M en asilos de ancianos. La población de estos asilos es susceptible a ITU y a tener complicaciones sistémicas; por ello, el objetivo del estudio fue determinar la frecuencia de genes de virulencia y la presencia del gen *bla*_CTX-M _en UPEC aisladas en residentes de asilos de ancianos ubicados en Lima Metropolitana.

**MENSAJES CLAVE**

Motivación para realizar el estudio: los asilos de ancianos, por la alta prevalencia de infecciones urinarias debido a *Escherichia coli *multidrogo resistente y el alto riesgo de generar urosepsis, merecen una especial atención epidemiológica.

Principales hallazgos: el 57,1% de las *E. coli* productoras de BLEE fueron portadores del gen *bla*_CTX-M_; de los cuales el 70% pertenecieron al gen *bla*_CTX-M-grupo 2_. Además, se obtuvo una frecuencia del 46% y 37% de *hly-alfa* y *cnf-1*, respectivamente; y, finalmente, una frecuencia del 46% y 91% del gen p*ap G II* y *nanA*, respectivamente.

Implicancias: este es el primer reporte de genes resistentes asociados con BLEE en *E. coli *uropatógenas identificado en asilos de ancianos en el Perú; lo que tiene gran relevancia por constituir una amenaza para la salud de los adultos mayores.

## EL ESTUDIO

### Diseño de estudio y población

Estudio descriptivo observacional realizado en el 2018, en donde se recolectaron de forma aleatoria 35 aislamientos no repetidos de *E. coli *productoras de BLEE obtenidas de muestras de orina de adultos mayores residentes de seis asilos de ancianos privados de Lima Metropolitana en Perú.

### Estudio microbiológico

La identificación bacteriana se realizó por pruebas bioquímicas convencionales (triple sugar iron agar, lysine iron agar, agar citrato, agar movilidad-indol-orinitina y rojo de metilo/Voges-Proskauer) y confirmadas molecularmente, por reacción en cadena de la polimerasa (PCR, por sus siglas en inglés), a través de la amplificación del gen *uspA*.

### Pruebas de susceptibilidad

Las *E. coli *productoras de BLEE fueron confirmadas por el método de disco difusión especificado en el documento M02-13 del Clinical & Laboratory Standards Institute (CLSI) ^8^; además, se determinó la susceptibilidad antimicrobiana por el método de disco difusión de acuerdo con los lineamientos del CLSI^ (^ ^8^, empleando como control de calidad a la *E. coli* ATCC 25922. Se incluyeron los discos de antibióticos con los siguientes antibióticos: piperacilina/tazobactam 100/10 µg (PTZ); amoxicilina/ácido clavulánico 20/10 µg (AMC); cefotaxima 30 µg (CTX); ceftazidima 30 µg (CAZ); cefepime 30 µg (FEP); cefoxitina 30 µg (FOX); aztreonam 30 µg (AZM); meropenem 10 µg (MEM); imipenem 10 µg (IMP); amikacina 30 µg (AK); gentamicina 10 µg (GM); ciprofloxacina 5 µg (CIP); nitrofurantoina 300 µg (NIT) y trimetoprim/sulfametoxazol 1,25/23,75 µg (SXT).

### Detección de genes de virulencia y resistencia

El ADN bacteriano se extrajo usando DNA Purification kit GeneJetGenomic (ThermoScientific), siguiendo las recomendaciones del fabricante. Se determinó la presencia de ocho genes de virulencia: *aer, hly, cnf-1, chuA, TcpC, nanA, pap GII*, *iucC* ^(^ ^9^ ^,^ ^10^ ^) ^y tres genes de resistencia asociados a BLEE: *bla*_CTX-M,_ *bla*_SHV_ y *bla*_TEM_^ (^ ^11^. Además, se realizó la detección de diferentes grupos de aislados portadores de *bla*_*CTX-M *_(1, 2, 8 y 9)^ (^ ^12^. Las condiciones de la PCR fueron llevadas a cabo en un volumen final de 30 μl que contenía 1X del MaximoTaq DNA (GeneON) así como 0,5 uM de cada uno de los cebadores y 1,5 mM de MgCl_2_. El volumen de ADN incorporado fue de 2,0 μL. El termociclador Labnet, Multigene optimax se programó con los siguientes parámetros: denaturación inicial a 94 °C por tres minutos; denaturación a 94 °C por 30 s; extensión a 72 °C por 30 s; en 30 ciclos de reacción y extensión final a 72 °C durante tres minutos, mediante el termociclador Labnet, Multigene optimax. En la Tabla 1 se muestra la secuencia de cebadores utilizados, el tamaño de los productos de amplificación, la temperatura de hibridación y la referencia del estudio. Los fragmentos de ADN amplificados fueron separados mediante electroforesis en gel de agarosa al 1% durante 50 min a 110 voltios. Finalmente, se usó el *buffer* de carga Runsafe (GeneON) para su revelado en UV.

**Tabla 1:** Genes de virulencia y resistencia, características de amplificación.

Gen	Secuencia de cebadores (5’ - 3’)	Producto (pb)	Temperatura de hibridación (°C)
Genes de virulencia			
α-hemolysin (α-*hly*)	AACAAGGATAAGCACTGTTCTGGCT	1177	63
	ACCATATAAGCGGTCATTCCCGTCA		
*chuA*	GACGAACCAACGGTCAGGAT	279	55
	TGCCGCCAGTACCAAAGACA		
Aerobactin synthesis (*aer*)	TACCGGATTGTCATATGCAGACCGT	602	63
	AATATCTTCCTCCAGTCCGGAGA AG		
Aerobactin synthesis (*iucC*)	CTCGAATTCACTGGGATTTGGTCAACC	1701	62
	CTCTCTAGAATTCCTGAGTTACCAGCC		
Cytotoxic necrotizing factor (*cnf1*)	AAGATGGAGTTTCCTATGCAGGAG	498	61
	CATTCAGAGTCCTGCC CTCATTATT		
P - fimbriae (*pap*)-* alelo II*	GGGATGAGCGGGCCTTTGAT	190	65
	CGGGCCCCCAAGTAACTCG		
*nanA*	ACCGGTGAGGGGAAATAAAC	216	59
	GGTGAGTACCAGGGCGATTA		
*tcpC*	GGCAACAATATGTATAATATCCT	386	51
	GCCCAGTCTATTTCTGCTAAAGA		
Genes de resistencia			
*bla*SHV	ATGCGTTATATTCGCCTGTG	544	58
	GTTAGCGTTGCCAGTGCTCG		
*bla*TEM	ATAAAATTCTTGAAGACGAAA	1080	54
	GACAGTTACCAATGCTTAATC		
*bla*CTX-M	TTTGCGATGTGCAGTACCACTAA	865	60
	CGATATCGTTGGTGGTGCCAT		
*bla*CTX-M-grupo 1	ATGGTTAAAAAATCACTG C	900	55
	GGTGACGATTTTAGCCGC		
*bla*CTX-M-grupo2	CGTTAACGGCACGATGAC	404	59
	CGATATCGTTGGTGGTGCCAT		
*bla*CTX-M-grupo8	ACGCTCAACACCGCGATC	490	63,3
	CGTGGGTTCTCGGGGATAA		
*bla*CTX-M-grupo9	GATTGACCGTATTGGGAGTTT	831	58
	CGGCTGGGTAAAATAGGTCA		

### Análisis estadístico

El análisis estadístico se realizó mediante el programa SPSS Statistics for Windows, versión 25.0. Armonk, NY: IBM Corp. La descripción de las variables cualitativas se realizó a través de gráficas de frecuencia. 

### Consideraciones éticas

El presente estudio obtuvo la aprobación del Comité de Ética de la Facultad de Medicina de la Universidad Nacional Mayor de San Marcos. Acta 1812 con código de proyecto 0013.

HALLAZGOS

Susceptibilidad antimicrobiana

Se analizaron 35 UPEC productoras de BLEE provenientes de asilos de ancianos. La mayoría de los aislamientos fueron resistentes a cefotaxima (25/35), no hubo aislamientos resistentes a carbapenémicos, y solo dos (2/35) aislamientos fueron resistente a cefoxitina. Además de la resistencia a los ß-lactámicos se pudo observar que las cepas de UPEC presentaron amplia resistencia a ciprofloxacina, 82,9% (29/35) de los casos. Asimismo, no se encontraron UPEC resistentes a PTZ, MEM e IMP. En la Figura 1 se detalla las frecuencias de resistencia por antibiótico.

**Figura 1:** Perfil de resistencia a los antimicrobianos de los aislamientos de *Escherichia coli *productoras de ß-lactamasas de espectro extendido (n=35).

### Genotipificación de UPEC productoras de* bla*_CTX-M_

De los 35 aislamientos, 57,1% (20/35) fueron portadores del gen *bla*_CTX-M_, de los cuales el 70% (14/20) correspondieron al *bla*_CTX-M-grupo 2_, 40% (8/20) al *bla*_CTX-M-grupo 1_, 30% (6/20) al *bla*_CTX-M-grupo 9 _y 20% (4/20) al *bla*_CTX-M-grupo 8_. Por otro lado, el 60% (12/20) de las UPEC portadoras del gen *bla*_CTX-M _tuvieron dos o más variantes alélicas del gen *bla*_CTX-M _en la misma bacteria. Además, hubo un mayor nivel de confluencia entre los genes pertenecientes al *bla*_CTX-M-grupo 1 _y *bla*_CTX-M-grupo 2_ (Tabla 2). También se evaluó la presencia de otros genes productores de ß-lactamasas (*bla*_*SHV*_ y *bla*_*TEM*_) en los productores y no productores de *bla*_CTX-M_. De un subestrato de las UPEC analizadas el 48,1% (13/27) no resultaron portadoras del gen *bla*_CTX-M_, pero sí a los genes *bla*_*SHV*_, y/o *bla*_*TEM *_. Asimismo, el 51,9% (14/27) de las UPEC presentaron al gen *bla*_CTX-M_, además de los genes *bla*_*SHV*_, y/o *bla*_*TEM*_. No se analizó las secuencias nucleotídicas de las variantes alélicas que confirmarían su pertenencia al grupo BLEE.

**Tabla 2:** Distribución de grupos del gen *bla*_*CTX-M*_ en las* Escherichia coli *uropatógenas productoras de ß-lactamasas de espectro extendido.

### Genotipificación de factores de virulencia en UPEC productoras de BLEE

De las 35 UPEC productores de BLEE se analizaron los genes relacionados con exotoxinas, en los cuales se obtuvo una frecuencia del 46% (15/35) y 37% (13/35) de *hly-alfa y cnf-1, *respectivamente. En los genes asociados al metabolismo del hierro se observó una elevada presencia de los genes *aer*, 94% (33/35) y *chuA*, 94% (33/35). Además, se evaluó la presencia de la adhesina *pap GII *obteniendo una frecuencia del 46% (16/34) y del gen *nanA *en el 91% (32/35) (Figura 2).

**Figura 2:** Frecuencia de genes de virulencia en *Escherichia coli *uropatógenas.

## DISCUSIÓN

Los resultados de este estudio estudio muestran que el 57,1% de las *Escherichia coli *productoras de BLEE presentaron el gen *bla*_CTX-M_, siendo el 70% del grupo *bla*_CTX-M-grupo 2_. Por otro lado, los genes: *hly-alfa y cnf-1, *codificantes a exotoxinas, se detectaron con una frecuencia del 46 y 37%, respectivamente.

Los asilos de ancianos son instituciones de larga estancia con una alta prevalencia de ITU ocasionado por bacterias multidrogorresistentes^ (^ ^3^. En este estudio, el 57,1% de las UPEC fueron portadoras del gen *bla*_CTX-M_. Este resultado es similar a los descritos por Galván *et al*.^13^ y Arce-Gil *et al*.^11^, quienes reportaron una frecuencia del 54,7% y 51,4%. Sin embargo, difieren de los descrito por Ramirez *et al*.^14^ ^) ^y Chavez^ (^ ^15^ ^) ^en diversos hospitales de Lima (70% y 91,7%). Esto último demuestra la gran variabilidad en la presencia de este gen en las instituciones de salud.

En lo referente a los resultados obtenidos sobre los subgrupos de *bla*_CTX-M_, fueron *bla*_CTX-M-grupo 2 _(14/20) seguido del *bla*_CTX-M-grupo 1 _(8/20) los de mayor presencia. Esto difiere de lo reportado por Chavez^ (^ ^15^, en donde no se detectó al *bla*_CTX-M-grupo 2 _y el *bla*_CTX-M-grupo 1_ (26/33) fue el de mayor frecuencia, y por Palma *et al*.^ (^ ^16^, donde predominó el *bla*_CTX-M-grupo 1_ (13/27). Sin embargo, estudios recientes indican que en Sudamérica *bla*_CTX-M-grupo 2 _es aún significativo^ (^ ^17^ y una reciente revisión en Brasil identifica a los *bla*_CTX-M-grupo 2 _y *bla*_CTX-M-grupo 1 _como las variantes más prevalentes en la región^ (^ ^18^.

También se evaluó la presencia de los genes *bla*_TEM _(54,3%) y *bla*_SHV _(51,4%); asimismo, se observó la presencia de los genes *bla*_TEM _y *bla*_SHV _entre las UPEC portadoras del gen *bla*_CTX-M_. Vale la pena resaltar que en el 37% de los aislamientos solo se identificó la presencia del gen *bla*_TEM _y/o *bla*_SHV_, y que, a pesar de que no se determinó su variante alélica y su dominio al grupo BLEE es un fuerte indicador de su pertenencia. Además, no se detectó la presencia de ninguno de los genes de resistencia evaluados en el 5,7% de los aislamientos, lo cual indica la presencia de otros genes de resistencia tipo BLEE no analizados en este estudio. 

Con relación con los factores de virulencia, el gen *pap GII *estuvo presente en el 47,1% de las UPEC. Este resultado difiere de lo reportado por Paniagua-Contreras *et al*. ^19^, en México, con un 21,1% de frecuencia del gen *pap G II* en UPEC comunitarias. También, se observó una alta frecuencia de genes asociación al transporte de hierro, similar a lo reportado por Dadi *et.al* ^20^, con 54,5% respecto al gen *iucC.*

Con relación a las exotoxinas estudiadas, obtuvimos una frecuencia del 44,2 y 38,2% de *hly-alfa y cnf-1, *respectivamente*, *resultados comparables al reportado por Dadi *et al.* ^20^ ^) ^con un 50,4 y 29% de *hly-alfa y cnf-1, *respectivamente. Por otro lado, en este estudio no encontramos UPEC portadoras del gen *tcpC*, a diferencia de otros trabajos en los que se han reportado prevalencias de hasta el 25% ^10^.

También encontramos una frecuencia del 88,2% para el gen *nanA*, el cual es importante en la producción energética. Se ha propuesto que la presencia del gen *nanA *genera una alta competitividad en las UPEC para generar bacteriemias, aunque su rol en la patogénesis de la ITU es menos importante^ (^ ^6^, los resultados indican la existencia de un alto riesgo en los residentes con bacteriemias de desarrollar sepsis.

Este estudio presenta algunas limitaciones. Si bien se realizó en varios asilos de ancianos, no se obtuvo información clínica de los pacientes, además de presentar un número limitado de muestras. Por otra parte, no se analizaron más genes de virulencia y resistencia (otros tipos de BLEE) relevantes en la epidemiología de las UPEC. 

En conclusión, este es el primer reporte de UPEC productoras de BLEE en asilos de ancianos en Perú; donde podemos observar el predominio del gen *bla*_CTX-M_, de los cuales el 70% pertenecen al *bla*_CTX-M-grupo 2_. También, se puede indicar que las UPEC productoras de BLEE en los asilos de ancianos presentan una elevada frecuencia de exotoxinas y del gen *nanA*, lo que les confiere una ventaja para diseminarse hacia el torrente sanguíneo. Esperamos que estos hallazgos permitan fortalecer la vigilancia epidemiológica de bacterias multirresistentes y prevenir su diseminación.
